# MuTCELM: An optimal multi-TextCNN-based ensemble learning for text classification

**DOI:** 10.1016/j.heliyon.2024.e38515

**Published:** 2024-09-30

**Authors:** Victor Kwaku Agbesi, Wenyu Chen, Sophyani Banaamwini Yussif, Chiagoziem C. Ukwuoma, Yeong Hyeon Gu, Mugahed A. Al-antari

**Affiliations:** a School of Computer Science and Engineering, University of Electronic Science and Technology of China, No. 2006, Xiyuan Ave, West Hi-Tech Zone, Chengdu, Sichuan, China; b Sichuan Engineering Technology Research Center for Industrial Internet Intelligent Monitoring and Application, Chengdu University of Technology, No. 610059, Chengdu, Sichuan, China; c Department of Artificial Intelligence and Data Science, College of AI Convergence, Daeyang AI Center, Sejong University, Seoul 05006, Republic of Korea

**Keywords:** Deep learning, Ensemble learning, Feature extraction, Multi-TextCNN, Text classification

## Abstract

Feature extraction plays a critical role in text classification, as it converts textual data into numerical representations suitable for machine learning models. A key challenge lies in effectively capturing both semantic and contextual information from text at various levels of granularity while avoiding overfitting. Prior methods have often demonstrated suboptimal performance, largely due to the limitations of the feature extraction techniques employed. To address these challenges, this study introduces Multi-TextCNN, an advanced feature extractor designed to capture essential textual information across multiple levels of granularity. Multi-TextCNN is integrated into a proposed classification model named MuTCELM, which aims to enhance text classification performance. The proposed MuTCELM leverages five distinct sub-classifiers, each designed to capture different linguistic features from the text data. These sub-classifiers are integrated into an ensemble framework, enhancing the overall model performance by combining their complementary strengths. Empirical results indicate that MuTCELM achieves average improvements across all datasets in accuracy, precision, recall, and F1-macro scores by 0.2584, 0.2546, 0.2668, and 0.2612, respectively, demonstrating significant performance gains over baseline models. These findings underscore the effectiveness of Multi-TextCNN in improving model performance relative to other ensemble methods. Further analysis reveals that the non-overlapping confidence intervals between MuTCELM and baseline models indicate statistically significant differences, suggesting that the observed performance improvements of MuTCELM are not attributable to random chance but are indeed statistically meaningful. This evidence indicates the robustness and superiority of MuTCELM across various languages and text classification tasks.

## 1 Introduction

Text classification is the process of categorizing text into structured groups, classes, or labels, which holds significant importance in both organizational and personal contexts. For example, user-centered applications can promptly provide users with accurate solutions by offering recommendations based on their preferences. Similarly, organizations can enhance their processes, improve data-driven operations, and assess textual systems to benefit stakeholders [1], [2]. However, text data is inherently complex, characterized by a wide array of linguistic nuances such as synonyms, antonyms, homonyms, and idioms, which complicates accurate classification. Languages such as Arabic, Ewe, and Urdu, for instance, contain words with multiple meanings (polysemy) depending on the context, further challenging classification models [3], [4], [5]. Moreover, text data often has high dimensionality due to the extensive vocabulary, leading to the “curse of dimensionality,” which makes models computationally intensive and difficult to train effectively. A critical factor in determining the efficiency of a classification model in text classification is the choice of feature extraction technique. In this context, feature extraction involves converting raw textual data into numerical feature representations that are used by models for classification tasks [5]. This process is of paramount importance to the research community and is essential for achieving optimal classification performance.

**Table:** 

**Nomenclature**			
AJGT	Arabic Jordanian General Tweets	MSA	Modern Standard Arabic
ALJ-News	Al-Jazeera-News	Word2vec	Word to Vector
BoW	Bag of Words	TF-IDF	Term Frequency-Inverse Document Frequency
GloVe	Global Vectors	LSTM	Long Short-Term Memory Network
CNN	Convolutional Neural Network	RNN	Recurrent Neural Network
DL	Deep Learning	ML	Machine Learning
OFDL	Optimized Fuzzy Deep Learning	EDL	Ensemble Deep Learning
TEL	Traditional Ensemble Learning	Multi-TextCNN	Multiple Text CNN
MuTCELM	Multi-TextCNN-based Ensemble Learning Model	NB	Naive Bayes
SVM	Support Vector Machine	GRU	Gated Recurrent Unit
EAdaCLE	Efficient Adaptive Convolutional-Based Label Embedding	ASA	Arabic Sentiment Analysis
SANAD	Single-Label Arabic News Articles Dataset	NADiA	News Articles Dataset in Arabic
BERT	Bidirectional Encoder Representations from Transformers	ALBERT	A Lite BERT
DeBERTa	Decoding-Enhanced BERT with Disentangled Attention	ReLU	Rectified Linear Unit

Previously, various methods such as Bag of Words (BoW), term frequency-inverse document frequency (TF-IDF), word embeddings (e.g., Word2Vec and GloVe), Convolutional Neural Networks (CNNs), Recurrent Neural Networks (RNNs), Long Short-Term Memory Networks (LSTMs), and transformer-based models have been employed for feature extraction from raw texts. However, these methods have faced several limitations [1], [6]. For instance, BoW and TF-IDF methods disregard word order and context, leading to a loss of semantic meaning, which is crucial in tasks like sentiment analysis. Standard Word2Vec and GloVe embeddings do not adequately address polysemy or context variations across different dialects. Traditional CNNs capture local dependencies in text data but often fail to account for long-range dependencies and deeper contextual information. RNNs are prone to vanishing gradient issues, which diminishes their effectiveness in capturing long-range dependencies. While transformer-based models offer improved performance, they are challenged by high computational costs and memory usage and still struggle with certain types of ambiguity and contextual nuances. Recent studies have explored alternative deep learning (DL) approaches that have demonstrated improved performance due to their advanced feature-learning capabilities. For example, Agbesi et al. [7] proposed a DL-based double-layer Bi-GRU method for extracting features from sentimental texts. This method uses Bi-GRU layers to produce semantic feature vectors, representing the texts in matrix form with a time-step dimension. Additionally, Yazdinejad et al. [8] introduced an optimized fuzzy deep learning (OFDL) model for data categorization, utilizing the Non-Dominated Sorting Genetic Algorithm II (NSGA-II). The OFDL model optimizes both DL and fuzzy learning in multi-modal contexts, finding optimal trade-offs between competing objectives by minimizing feature numbers and maximizing feature weights. OFDL employs Pareto-optimal multi-objective optimization using NSGA-II, enhancing backpropagation and fuzzy membership functions. The fusion layer of OFDL combines enhanced views of DL with fuzzy learning, producing high-level representations and optimal features for classification tasks characterized by significant uncertainties and noise. However, this approach is challenged by increased complexity due to the integration of fuzzy logic with DL, necessitating further optimization techniques. Ensemble deep-learning (EDL) techniques have also been developed to enhance classification performance. Typically, DL models are integrated into traditional ensemble learning (TEL) techniques to form EDLs. These approaches have demonstrated that combining DL with ensemble methods surpasses the performance of baseline DL models. For example, Wu et al. [9] constructed various base learners with differing capacities for generalization and label correlation exploration, subsequently combining them with a bagging technique to improve model robustness. Another study introduced a weighted ensemble technique that learns the weights of base learners and integrates them for optimal classification performance [10]. Moreover, researchers have employed a boosting-based ensemble technique to adjust the weights of base classifiers and their data distribution, thereby improving performance in downstream tasks [11].

Despite advances in these methods, many models still struggle to fully grasp nuanced contexts, mainly when dealing with complex or polysemous words, and are often impractical for resource-constrained environments due to high computational and memory requirements. Models trained on specific datasets frequently exhibit poor generalization to other domains or languages, leading to suboptimal performance. Additionally, preset parameters pose challenges to achieving optimal text classification for languages such as Ewe, Arabic, and Urdu, which have complex morphologies and semantic representations. Improving text classification across multiple languages, including Ewe, Arabic, and Urdu, is critical for several reasons. Firstly, accurate text classification is vital due to the inherent complexity of text data, which encompasses linguistic nuances like synonyms, antonyms, homonyms, and idioms. For languages with polysemous words, effective classification models must accurately interpret context to avoid confusion. This complexity underscores the need for models capable of handling diverse linguistic features and performing effectively across various languages and contexts [12]. Secondly, the high dimensionality of text data, driven by extensive vocabulary sizes, results in computationally intensive models. Therefore, efficient feature extraction techniques are essential for converting raw text into numerical data that models can process efficiently. Models trained on specific datasets often fail to generalize well across different domains or languages. By enhancing text classification techniques, models can better manage diverse linguistic features and maintain robust performance across different languages and contexts [13]. Thirdly, improving text classification models for languages with complex morphologies and semantic representations, such as Ewe, Arabic, and Urdu, promotes technological inclusiveness and versatility, enabling AI-driven solutions to be more broadly applicable and practical. This is particularly significant as multilingual text classification tools are increasingly used to automate business processes, leading to increased productivity and reduced costs. Lastly, accurate and efficient text classification models can substantially impact real-world applications, including sentiment analysis, news detection, and recommendation systems, thereby streamlining processes and enhancing data-driven operations for organizations [12], [14].

Motivated by the need for an optimal classification model that addresses overfitting and the lack of sufficient syntactic and semantic features in Ewe, Arabic, and Urdu texts, this study proposes MuTCELM, a Multi-TextCNN-based ensemble learning model designed to achieve optimal performance in multi-class text classification across different datasets. MuTCELM employs five sub-classifiers, each of which is a variant of the proposed Multi-TextCNN, tailored to capture different granularity of text data. These sub-classifiers are then integrated into an ensemble framework where their outputs are combined using weighted averaging to produce the final classification result. Unlike previously introduced methods, MuTCELM addresses existing limitations by utilizing Multi-TextCNN and transformer-based models to improve contextual understanding at various granular levels, enhancing the handling of polysemy and contextual nuances. Additionally, MuTCELM's weight-averaging approach reduces computational costs and improves efficiency, making it practical for large-scale applications. Consequently, MuTCELM offers richer and more comprehensive text classification across diverse datasets. The contributions of this study are as follows:

1. Proposed an enhanced feature extraction model, Multi-TextCNN, capable of capturing high semantic features and relevant patterns and relationships indicative of class membership.
2. Using the proposed model as a network backbone, analysis of five different sub-classifiers was performed for multiclass text classification across different tasks and languages.
3. A comprehensive experiment is conducted to compare and evaluate MuTCELM's performance against baseline models, different levels of model combinations, and established ensemble methods.

This study's objectives are highlighted as follows:

• To design and implement MuTCELM, a Multi-TextCNN-based Ensemble Learning Model optimized for text classification tasks across various languages. The model aims to integrate multiple sub-classifiers to leverage their complementary strengths for improved classification accuracy.
• This study seeks to enhance the performance of text classification by optimizing the ensemble learning framework. The goal is to achieve superior accuracy, precision, recall, and F1-macro scores compared to existing text classification models.
• To evaluate the effectiveness of the proposed MuTCELM across multiple languages, including less-resourced languages like Ewe, as well as widely spoken languages such as Arabic and Urdu. The study aims to demonstrate the model's adaptability and robustness in handling diverse linguistic datasets.
• Finally, the study aims to explore and apply optimization techniques within the ensemble learning framework to ensure that MuTCELM not only improves performance but also maintains computational efficiency and scalability.

The study is structured as follows: Section 2 introduces the current related studies; Section 3 describes materials and the proposed model; and Section 4 presents and analyzes the results. Also, the effects of the proposed MuTCELM are discussed. Lastly, the study is concluded in Section 5.

## 2 Related work

Previously, the deployed feature extraction methods were mainly based on TF-IDF and N-grams, coupled with basic implementations of Naive Bayes (NB) and support vector machine (SVM) models. Farhoodi et al. Farhoodi et al. [15] developed a Persian text categorization model based on text-level N-grams, while other studies enhanced TF-IDF for specific downstream tasks [16]. Feature selection has been a focus, with multiple studies evaluating various strategies and machine learning (ML) approaches to improve classification performance [17], [18]. However, these methods have limitations in capturing the full context and deep semantic information from texts. With advancements in neural networks, DL models such as RNNs, LSTM networks, and Gated Recurrent Units (GRUs) have become prominent in text classification. These models offer improved capabilities in representing data and extracting complex features. Various studies have employed different neural architectures for text classification tasks. For example, a collaborative training approach with RNNs was proposed for text categorization, highlighting the potential of DL models in handling text data more effectively [19]. CNNs have also been widely adopted for text representation and classification. Kim's CNN model introduced convolutional operations at various levels to derive text representations, leading to several successful applications in text classification [20]. Subsequent studies combined CNNs with other architectures, such as GRUs and LSTMs, to further enhance performance. For instance, a CNN-GRU model was applied to the NADIA and SANAD datasets for Arabic text classification, demonstrating the versatility of CNN-based methods in different contexts [1]. Recently, hybrid models that integrate multiple neural network architectures have gained attention for their ability to capture diverse features and to improve classification accuracy. For example, a CNN-LSTM technique was utilized for Arabic sentiment analysis, showing robust performance across multiple datasets [21]. Agbesi et al. [7] developed an attention-based double-layer Bi-GRU model to analyze sentiments. Similarly, the EAdaCLE model employed adaptive convolutional techniques with label embedding for multilingual text classification, indicating the growing interest in models that can handle multiple languages and domains effectively [14]. Despite these advancements, existing models often face challenges in resource-constrained environments and fail to generalize well across different languages and domains.

Vaswani et al. [22] recently introduced transformers designed to compute the similarity between word vectors. This development paved the way for the introduction of a groundbreaking natural language representation model known as BERT (Bidirectional Encoder Representations from Transformers) [23]. BERT is designed to pre-train deep bi-directional representations from extensive corpora, marking a significant advancement in text classification. This led to the development of a bidirectional encoder-based approach for Russian-text classification [24]. Similarly, a study introduced EweBERT for a downstream task [25]. Results indicated that the proposed EweBERT outperformed benchmark machine learning methods. Also, MARBERT and ARBERT, both BERT-based models, were trained on a vast sample of Arabic text data, specifically one billion Arabic tweets [26]. These tweets were selected by randomly sampling content from a substantial proprietary dataset, which included nearly 6 billion tweets, a staggering 15.6 billion tokens, and sequences of up to 128 characters in length. The authors harnessed the MARBERT model to address the challenging task of ASA. Ensemble learning has earned its reputation as one of the most pivotal and impactful techniques. This is because of the increasing heterogeneity among standard classifier groups, diverse ensemble techniques, and sub-sampling or cross-validation (creating multiple datasets from the original dataset). Ensemble methods seek to improve the accuracy of predictions by combining probabilities from multiple sub-models into a single model. In addition, they minimize variance and biases, prevent overfitting, and mitigate the challenges of baseline models [27]. These methods have been deployed with several downstream tasks, including sentiment analysis, news classification, sarcasm, and fake news detection. For instance, Akhtyamova et al. [28] introduced a CNN-based voting ensemble technique for predicting drug safety based on patients' feedback.

Recently, techniques, including LSTM, CNN, and GRU, are combined into an ensemble utilizing a voting strategy. For instance, Heikal et al. [42] utilized the LSTM and CNN models as minor classifiers and then integrated their predictions into a voting-based ensemble method. Al-Omari et al. [45] proposed a similar voting-based deep ensemble method applied to the NLP4IF-2019 dataset. The authors in [46] suggested a multi-label-based ensemble framework adopting stacking and voting ensemble techniques on both the Toxic Posts and Semeval2018-Task datasets. Other proposed studies have compared the superiority of EDL methods. For example, Mohammed and Kora suggested a meta-ensemble DL method to boost the accuracy of user opinions using a new Arabic-Egyptian-V2 [4]. Similarly, [3] compared different ensemble approaches on six public datasets, including voting, stacking, and meta-learning. Their results demonstrate that ensemble approaches significantly improve classification results. The study of El Karfi and El Fkihi [50] ensemble XLM-T (a multi-lingual model) and MARBERT (a mono-lingual model) to solve the intricacies of the Arabic language, which are challenging for single models. Also, a study represents tweets using hybrid features, which combine syntactic and semantic information [5]. Their syntactic information is obtained using the BoWs, while semantic information is derived from the fastText-based and domain-specific methods. Then, a multi-channel CNN model ensembles CNNs to collect multi-scale information for a Nepali-based classification task. However, their model recorded a lower accuracy score of 0.713. From the above literature, the study identified that most methods suffer from overfitting due to simplistic feature representations, leading to insufficient high-level feature extraction. In addition, few employ a traditional single-level kernel to extract features of relations between words or documents using single classifiers, leading to unsatisfactory classification performance. Also, some of the studies only fine-tuned BERT using the Arabic datasets for a specific task, taking advantage of the self-attention mechanism in the BERT-based models. The model proposed in this study aims to address these limitations by leveraging a Multi-TextCNN-based ensemble learning approach. This model integrates CNNs with transformer-based models to enhance contextual understanding and reduce computational costs, making it suitable for large-scale applications and diverse linguistic contexts. A detailed summary of other studies employing different ensemble methods, classifiers, and datasets is presented in Table 1.

**Table 1:** Summary of other ensemble learning methods for multiclass text classification.

Previous study	Method	Classifier	Classification task	Dataset
Xia et al., (2016) [29]	Voting	SVM, LR	Sentiment Analysis	Amazon text
Onan et al., (2016) [30]	AdaBoost, Stacking, Bagging	BLR, NB, LDA, LR, SVM	Sentiment Analysis	Tweets
Ankit et al., (2018) [31]	Voting	LR, RF, SVM, NB	Sentiment Analysis	Tweets
Oussous et al., (2018) [32]	Stacking, Voting	ME, MNB, SVM	Sentiment Analysis	Moroccan tweets
Pasupulety et al., (2019) [33]	Stacking	RF, SVM	Sentiment Analysis	Indian tweets
Seker et al., (2019) [34]	Bagging	RF, LR	Text Classification	Product reviews
Erdogan et al., (2019) [35]	voting, Stacking	SVM	Text Classification	Product reviews
Alrehili et al., (2019) [36]	Boosting, Bagging, Voting	SVM, NB	Sentiment Analysis	Client reviews
Cai et al., (2020) [37]	Voting	SVM, LR	Text Classification	Product reviews
Saeed et al., (2022) [38]	Voting, Stacking	SVM, NB, LR, DT, KNN	Sentiment Analysis	Arabic Corpus
Xu et al., (2016) [39]	Voting	LSTM, CNN	Sentiment Analysis	SemEval
Deriu et al., (2016) [40]	Stacking	CNN	Sentiment Analysis	SemEval
Araque et al., (2017) [41]	Voting, Stacking	GRU, CNN, LSTM	Sentiment Analysis	Movie reviews
Akhtyamova et al., (2017) [28]	Voting	CNNs	Sentiment Analysis	Medical reviews
Heikal et al., (2018) [42]	Voting	CNN, LSTM	Sentiment Analysis	ASTD
Akhtar et al., (2022) [43]	Voting, Stacking	LSTM, CNN, GRU	Sentiment Analysis	Twitter corpus
Minaee et al., (2019) [44]	Voting	CNN, LSTM	Sentiment Analysis	IMDB, SST2
Al-Omari et al., (2019) [45]	Voting	Bi-LSTM	Text Classification	Fake News
Haralabopoulos et al., (2020) [46]	Voting, Stacking	CNN, LSTM, RCNN, GRU, DNN	Sentiment Analysis	Semeval2018, Toxic comment
Livieris et al., (2021) [47]	Voting, Bagging, Stacking	Bi-LSTM, LSTM	Text Classification	Product reviews
Mohammadi et al., (2021) [48]	Stacking	Bi-LSTM, GRU, LSTM, CNN	Sentiment Analysis	SemEval-2016
Song et al., (2021) [49]	Stacking	SVM	Sentiment Analysis	ArSarcasm-v2
Mohammed et al., (2022) [3]	Voting, Stacking, Bagging	LSTM, LSTM-CNN, CNN, GRU, BLSTM-CNN, and GRU-CNN	Sentiment Analysis	Arabic Corpus, IMDB review, COVID19-Fake, SemEval, AJGT, ArSarcasm
El Karfi et al., (2022) [50]	SUM	AraBERT, CAMeLBERT	Sentiment Analysis	Twitter corpus, ASTD
Mohammed et al., (2023) [51]	Voting	XLM-T, MARBERT	Sentiment Analysis	ASAD, ArSarcasm-v2, SemEval
Kora et al., (2023) [4]	Stacking	GRU, LSTM, CNN	Sentiment Analysis	Arabic-Egyptian corpus
Sitaula et al., (2024) [5]	Fusion	CNNs (1,2,3,4)	Sentiment Analysis	NepCOV19Tweets

## 3 Materials and methodology

This section discusses the benchmark datasets in sub-section 3.1, the data preprocessing procedure in sub-section 3.2, and the proposed model in depth (see sub-section 3.3), specifically addressing how it fulfills the objective of outperforming state-of-the-art models.

### 3.1 Benchmark datasets

In this study, we employed datasets from three distinct languages—Ewe, Arabic, and Urdu—to evaluate the performance of MuTCELM.

The Ewe dataset [52] consists of news samples in the Ewe language obtained from Nigeria, Benin, Togo, Ghana, and Liberia. This dataset includes 4264 news items categorized into six distinct groups: sports, business, coronavirus, entertainment, political, and local news. The Arabic datasets comprise the Arabic Jordanian General Tweets (AJGT) dataset [53], Al-Jazeera-News (ALJ-News) dataset [54], and ArSarcasm-V2 dataset. The AJGT [53] dataset originated in 2017, with 1800 tweets annotated to classify sentiments as positive or negative. The AJGT is an Arabic language that encompasses MSA and Jordanian dialects. The ALJ-News [54] datasets contain news articles in Arabic extracted from the aljazeera.net (Arabic news portal) website. It comprises five (5) Arabic classes, including arts, economics, science, politics, and sports, all totaling 1500 news articles. ArSarcasm-V2 [55] is an extension of the original dataset ArSarcasm created as a result of combining ArSarcasm with DAICT, a corpus consisting of 5358 tweets written in Modern Standard Arabic (MSA), colloquial Arabic, and a selection of baselinely collected tweets. Each tweet contains elements of sentiment, sarcasm, and dialect. To provide a diverse range of dialects, the dataset comprises five distinct dialects: 10885 tweets from MSA, 2981 tweets from Egyptian Arabic, 966 tweets from the Gulf region, 671 tweets from the Levant area, and 45 tweets from the Maghreb region. The final dataset consists of 15548 tweets. The Urdu corpus [56] contains fake and legitimate news on FakeNewsAMT and celebrity classes. Each article was acquired from several US-based websites, including CNN, ABCNews, NewYorkTimes, FoxNews, USAToday, CNET, Bloomberg, and others. These datasets were chosen to test the multilingual capabilities of our model and to ensure robustness across different linguistic structures. Detailed descriptions of these corpora are described in Table 2.

**Table 2:** Details of benchmark datasets.

Dataset	Language	No. of Class	Task	Total
AJGT [53]	Jordanian dialects, MSA	2	Sentiment analysis	1800
ALJ-News [54]	Arabic dialect	5	News classification	1500
ArSarcasm-V2 [55]	Five Arabic dialects	3	Sentiment analysis	15548
Ewe [52]	Ewe language	6	News classification	4264
Urdu Corpus [56]	Urdu language	2	Fake news detection	900

### 3.2 Data preprocessing and embedding

The preprocessing procedure consists of two primary steps: word segmentation and stop-word removal. Because character-granular feature representation will significantly lose N-gram data, word-granular feature representation is used. There is no space between Arabic and Urdu words; word segmentation is required to separate these words. We skip this step for Ewe texts and use spaces as dividers. Furthermore, the stop word removal method eliminates several function words, such as prepositions and conjunctions. These function words do not contain deep semantic data, and their presence may even create noise, lowering classification performance indirectly. As a result, these words are removed from the preprocessing stage to increase its efficiency.

The preprocessed data is transformed into a dense vector of equal dimension. In contrast to the static word embedding technique, which assigns a word to a vector by directly referencing a word representation table, the study operates by taking an entire sentence as input and utilizing the hidden state of either the second-to-last hidden layer or the last hidden layer as the dynamic vector representation for all words within the sentence. Hence, a word's vector representations vary across contexts, conveying more precise semantic meanings. Given a word segmentation process that yields *α* words, it generates word vectors of dimension, *ρ*, with a dictionary matrix $B\in R^{\left(\alpha \times \rho \right)}R$ [23]. Let $\omega_{i}$ represent the $i^{th}$ word inside the phrase, and **W** denotes the input sentence. Let $x\in R^{\rho }$ represent the *ρ*-dimensional word vector for word $\omega_{i}$. The embedding procedure is used to create a text matrix $X\in R^{\left(l\times \rho \right)}$ by padding the sentences of each sentence, where *l* is the length of the padding sentence. The padding procedure ensures that all sentences are captured at equal length and then fed into the sub-classifier as input text.

### 3.3 Proposed MuTCELM

**Figure 1:**
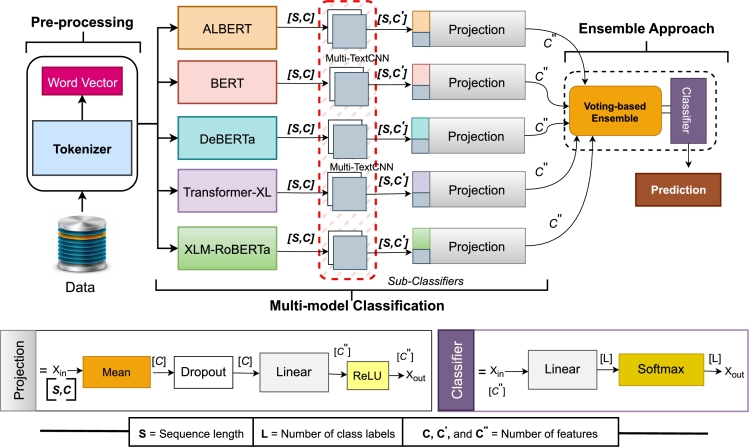
The proposed MuTCELM for multi-text classification tasks.

DL models such as CNN and LSTM are widely recognized for their hierarchical learning and automated feature extraction capabilities. However, optimizing text classification with transformer-based models remains challenging due to their fixed number of parameters. To address this challenge, a novel model that outperforms existing transformers while reducing computational time is necessary. To this end, we propose MuTCELM, a Multi-TextCNN-based Ensemble Learning Model optimized for text classification across various languages. MuTCELM integrates a new Multi-TextCNN model with transformer-based models, including ALBERT, BERT, DeBERTa, Transformer-XL, and XLM-RoBERTa, to generate five distinct sub-classifiers, weighted for different text classification tasks. These transformer-based models are implemented according to the principles outlined in their original studies. Each sub-classifier performs a unique function within the text classification task. The sub-classifiers differ in convolutional filter sizes, pooling strategies, and input feature representations. The outputs of these sub-classifiers are then aggregated using a voting mechanism, with the final classification decision determined by a weighted average of the sub-classifier outputs, as depicted in Fig. 1. We trained and evaluated MuTCELM using Ewe, Arabic, and Urdu datasets, as described in sub-section 3.1. For each language-specific dataset, we applied the preprocessing steps detailed in sub-section 3.2 to ensure compatibility with our model. In this study, the self-attention mechanism of each transformer-based model extracts different deep contextual information between texts and generates a feature matrix. Then, multiple convolutional kernels in the CNN are deployed to reinforce the relationships between texts. Finally, intricate and conceptual high-level features are extracted at various levels of granularity, enhancing the effectiveness of the classification model. Due to the preference parameter configuration challenge faced when training these transformer-based models, we employ a voting ensemble technique to compute the final output. The details of each component in the proposed MuTCELM are described below.

#### 3.3.1 Proposed multi-TextCNN

**Figure 2:**
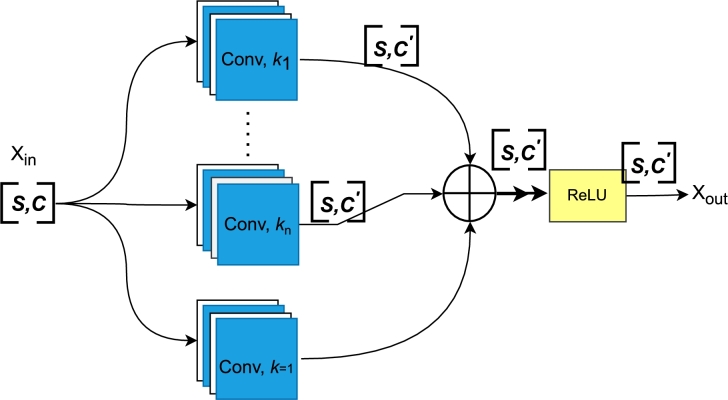
Proposed Multi-TextCNN. NOTE: *k*_*n*_ denotes the kernel size for the convolution. For *k* = 1 in a convolution, it denotes a residual branch.

This study proposes a new Multi-TextCNN (Multi-Text Convolutional Neural Network) that uses multiple convolutional kernels to reinforce the relationship between each text feature and obtain complex and abstract high-level features to enhance text classification performance. The proposed Multi-TextCNN, as shown in Fig. 2, is a robust, efficient, and optimized model for capturing text relationships. It is also introduced to capture the interactions and dependencies between texts, which is crucial for our study, where understanding how different documents relate to each other is essential. The Multi-TextCNN considers the entire content of multiple documents rather than just baseline sentences. Unlike traditional sequential models, the Multi-TextCNN processes multiple texts in parallel. This is particularly beneficial when dealing with tasks where data from multiple sources or documents is essential for making a classification decision. For instance, in sentiment analysis and fake news detection, the Multi-TextCNN comprises multiple parallel convolutional layers with different kernel sizes that capture different levels of n-gram features in the text. It is built based on the magnitude of the convolution kernel in the CNN, which is the same as the dimension of the word embedding vector. Assuming 768 is the computed size of the word vector, the convolution kernel is fixed at (3,768). As a result, whenever the convolution is performed with a stride of 1, the convolution kernel picks three different adjacent word vectors for the convolution process. The CNN algorithm extracts features from the sample with varying lengths by configuring multiple convolutional kernel-size parameters with diverse lengths. The mathematical formulation of Multi-TextCNN, as depicted in Eq. (1) is given below:

(1) $$M(x)=Relu(C_{k=1}(x)+C_{k_{1}}(x)+...+C_{k_{n}}(x)),$$

where $M(x)\in R^{S\times C}$, $C_{k}(\cdot )\in R^{S\times C^{\prime }}$ denotes a 1D convolution module with a *k* kernel size, and $x\in R^{S\times C}$ is the input feature. Table 3 shows the hyperparameter setting for our proposed Multi-TextCNN model.

**Table 3:** Summary of hyperparameter values.

Hyperparameter	Description
Number of Filters	128
Number of Conv. layers	3
Dense unit	128
Activation function	ReLU
Optimizer	Adam
Filter/Kernel size	[3,5,7]
Fusion method	Add

#### 3.3.2 Multi-TextCNN kernel sizes

As described above, the Multi-TextCNN leverages convolutional kernels of different sizes to extract features from text samples at various granularity levels. As specified in Table 3, using kernel sizes [3, 5, 7] allows the model to capture diverse patterns and dependencies within the text. For instance, the kernel size 3 is effective in capturing trigrams, which are sequences of three consecutive words. This is useful for detecting local patterns and short phrases, which often carry significant contextual information. In the sentence “Ele fu kpem le dɔléle sesẽ ade ta,” the kernel size 3 captures patterns like “Ele fu kpem,” “fu kpem le,” and “kpem le dɔ.” These short sequences help identify subjects, actions, and objects in the text. Similarly, kernel size 5 captures five-gram sequences, providing a broader context than trigrams (i.e., kernel size 3), which recognizes slightly longer dependencies and more complex relationships between words. In contrast, kernel size 7 captures even longer sequences, such as seven-grams, which are useful for understanding long-term dependencies and overarching themes within the text. Combining kernel sizes [3, 5, 7] enables multi-scale feature extraction, capturing features at different granularities simultaneously. This leads to a richer and more comprehensive feature set, enhancing the model's robustness and generalization. Consequently, the model better handles and understands Ewe, Arabic, and Urdu text structures and lengths, improving overall performance.

#### 3.3.3 Multi-modal classification

During the multi-modal classification phase, five Multi-TextCNN-based sub-classifiers are constructed after the initial data pre-processing. These sub-classifiers are constructed by combining the proposed Multi-TextCNN with each transformer-based model. In this study, the sub-classifiers are trained as follows: first, a multi-layer encoder is used to create a semantic feature representation of the text after the pre-classified training set is inputted into each model. Second, the newly proposed Multi-TextCNN convolutional layer receives the final hidden state *h* of each model *i* after it is removed. Lastly, multiple kernels are utilized to extract high-level text feature vectors, which leads to additional feature representations between texts. Let *X* denote the input text. The encoder *E* transforms *X* to produce a hidden state representation $H_{i}$ for each model *i* as shown in Eq. (2):

(2) $$H_{i}=E_{i}(X),$$

where $E_{i}$ represent the encoder for model *i*. After encoding, the final hidden state $h_{1}$ of each model *i* is extracted from $H_{i}$ as shown in Eq. (3):

(3) $$h_{i}=H_{i}[-1],$$

where $H_{i}[-1]$ is the final hidden state (i.e., last layer output) of the encoder $E_{i}$. This final hidden state $h_{i}$ is then fed into the newly proposed Multi-TextCNN convolutional layer. The Multi-TextCNN layer employs multiple convolutional kernels to extract high-level features from the final hidden state $h_{i}$. Let $k_{j}$ represent the j−th convolutional kernel with a kernel size [3,5, or 7]. The feature maps $F_{i,j}$ generated by applying $k_{j}$ on $h_{j}$ as shown in Eq. (4):

(4) $$F_{i,j}=conv(h_{i},k_{j}),$$

where $conv(h_{i},k_{j})$ denotes the convolution operation of kernel $k_{j}$ on the hidden state $h_{i}$ and $F_{i,j}$ represents the feature map obtained from the j−th kernel applied to the final hidden state $h_{j}$. For each hidden state $h_{j}$, we apply multiple convolutional kernels with sizes [3,5,7] to capture diverse features. This produces corresponding feature maps as: $F_{i,3}=[conv(h_{i}],k_{3})$, $F_{i,5}=conv(h_{i},k_{5})$, and $F_{i,7}=conv(h_{i},k_{7})$, respectively. We later applied max-pooling to each feature map $F_{i,j}$ to reduce its dimensionality and retain the most salient features. The pooled features $\left(p_{i,3},p_{i,5},p_{i,7}\right)$ are then concatenated in Eq. (5) to form the final feature representation for each text input as:

(5) $$f_{i}^{\prime }=concat(p_{i,3},p_{i,5},p_{i,7}).$$

In this study, the concatenated feature representation $f_{i}^{\prime }$ in Eq. (5) is then passed through a fully connected layer to produce the final classification output for each sub-classifier as shown in Eq. (6):

(6) $$\beta_{i}=\theta (f_{i}^{\prime }),$$

where *θ* represents the fully connected layer and activation function and $\beta_{i}$ is the predicted class probabilities (final output) of the sub-classifier for model *i*.

#### 3.3.4 Ensemble approach

Baseline models often have distinct parameters or structures, which can lead to inconsistency, bias, overfitting, complexity, and compatibility issues when preference parameters are set independently. To address these challenges, it is advantageous to employ approaches that enable shared preferences or joint optimization of parameters across models. For that, the study utilizes an ensemble technique to compute predictions based on the outcomes of each model, ensuring consistency, fairness, and interpretability. Ensemble learning, which involves aggregating multiple models such as classifiers, aims to improve classification and prediction accuracy while reducing the risk of erroneous predictions [57]. This is particularly important in text classification tasks, including sentiment analysis, fake news detection, and news classification, where the complexity and intricacies of the data are difficult to address with a single model. In this study, the final classification result is denoted as $\beta_{\pi }=[\beta_{1},\beta_{2},\beta_{3},\beta_{4},\beta_{5}]$, where $\beta_{i}$ represents the predicted class probabilities calculated by the sub-classifiers as described in Eq. (6). The approach integrates the outputs of five sub-classifiers, each contributing to the final prediction based on assigned weights that reflect their relative performance. This weighted voting ensemble technique ensures that more reliable sub-classifiers have a more significant influence on the final prediction, thereby enhancing the accuracy and robustness of the ensemble model. Specifically, each sub-classifier $\beta_{i}$ makes a prediction based on its own training and learned parameters. The sub-classifier's vote is assigned a weight $w_{i}$ based on its performance. The weight reflects the reliability and effectiveness of the sub-classifier. In this study, the weights are computed using cross-validation, where the performance of each sub-classifier is evaluated accordingly. The final predictions of the sub-classifiers $\beta_{1},\beta_{2},\beta_{3},\beta_{4},\beta_{5}$ are combined using their respective weights. The weighted sum of the votes V(c) for each class label $c_{i}$ is computed as shown in Eq. (7):

(7) $$V(c)=\sum_{i=1}^{n}w_{i}\cdot I(\beta_{i}=c)$$

The class label with the highest weighted vote is selected as the final prediction. This ensures that the sub-classifiers with higher reliability have a greater influence on the final decision. The final prediction, $\beta_{final}$ is formulated in Eq. (8) as follows:

(8) $$\beta_{final}=argmaxV(c)$$

The result is fed into a Softmax(⋅) activation function for a multi-classification task as shown in Eq. (9):

(9) $$\beta_{output}=Softmax\left(\beta_{final}\right).$$

### 3.4 Evaluation metrics

Four widely used evaluation metrics—recall, F1-macro, precision, and accuracy—were deployed, and an average performance was computed to evaluate the performance of our model. These metrics are extensively employed to assess the classification's performance. They are statistically computed in Eqs. (10) - (13) as follows:

(10) $$Accuracy=\frac{TP+TN}{TP+TN+FP+FN}$$

(11) $$Precision=\frac{TP}{TP+FP}$$

(12) $$Recall=\frac{TP}{TP+FN}$$

(13) $$F1-macro=\frac{1}{n}\sum 2\times \frac{Precision\times \mathrm{Recall}}{Precision+\mathrm{Recall}}$$

where TP (True positive) denotes the correct prediction of positive samples, TN (True negative) represents the accurate prediction of negative samples, and FP (False positive) indicates the incorrect prediction of negative samples as positive. Lastly, FN (False negative) signifies the inaccurate prediction of positive samples as negative.

### 3.5 MuTCELM training setting

First, a standard pre-trained word embedding is applied to each dataset. To enhance the parameters of our model, we use the Adam optimizer, with a learning rate of 1e−4 and a batch size of 16, which is appropriate for the sample size of each dataset. ReLU is employed as the activation function and set the convolution kernel to [3,5,7], each having 64 counts. The MuTCELM integrates predictions from five sub-classifiers using a weighted average of their output probabilities. The experimental setup involved benchmarking against five datasets selected for their varied linguistic characteristics. Experiments were conducted using a single RTX 3060Ti in a PyTorch-DL framework.

## 4 Results, analysis & discussion

This section presents a comparative analysis of MuTCELM's performance against state-of-the-art models using the AJGT, ALJ-News, ArSarcasm-V2, Ewe, and Urdu datasets. Specifically, sub-section 4.1 presents a detailed analysis of the results obtained for each dataset (see Table 4– Table 8), while sub-section 4.2 validates these findings through statistical analysis. The statistical analysis consists of the confidence intervals of each model provided to confirm the reliability of their mean performance on the datasets (see Table 9) and a statistical significance test to validate the performance improvements achieved by MuTCELM (see Table 10). Additional analysis, including the effects of varying parameters, is discussed in sub-section 4.3, depicted in Fig. 8. Furthermore, the results of MuTCELM are compared to those of baseline and ensemble-based techniques in Table 13. Finally, sub-section 4.4 provides a comprehensive discussion of the proposed model. In this study, ELM refers to the working principles of the ensemble of baseline models, excluding the Multi-TextCNN component.

**Table 4:** Results on AJGT dataset.

Type	Model	Accuracy	Precision	Recall	F1-macro	AVG
	ALBERT	0.679	0.674	0.661	0.667	0.670
	BERT	0.831	0.814	0.827	0.820	0.823
	DeBERTa	0.772	0.769	0.754	0.761	0.764
Baseline	Transformer-XL	0.763	0.758	0.761	0.759	0.760
	XLM-RoBERTa	0.806	0.798	0.789	0.793	0.796
	ELM	0.919	0.917	0.916	0.916	0.917

	ALBERT + Multi-TextCNN	0.799	0.792	0.787	0.789	0.791
	BERT + Multi-TextCNN	0.853	0.844	0.836	0.840	0.843
Effects of Multi-TextCNN	DeBERTa + Multi-TextCNN	0.869	0.850	0.866	0.858	0.860
	Transformer-XL + Multi-TextCNN	0.822	0.811	0.820	0.815	0.817
	XLM-RoBERTa + Multi-TextCNN	0.844	0.836	0.841	0.838	0.839
	**MuTCELM (Proposed)**	0.930	0.925	0.929	0.927	0.927

### 4.1 Results and analysis

#### 4.1.1 Results on AJGT dataset

**Figure 3:**
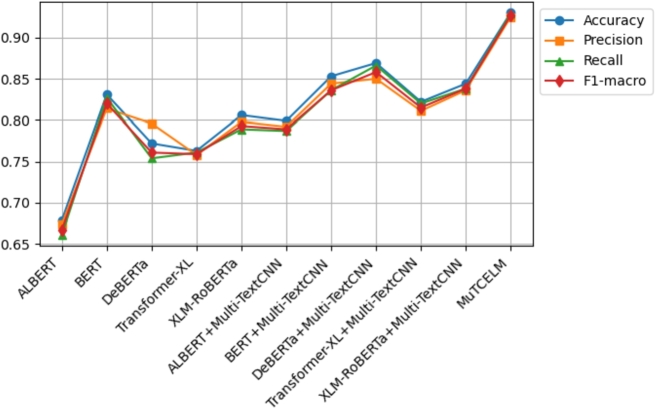
Summary of models performance on AJGT dataset.

The results on the AJGT dataset achieved an accuracy of 0.714 [53]. However, in this study, experimental findings indicate that the standalone BERT model outperformed other compared models, achieving an accuracy of 0.831. Additionally, the DeBERTa + Multi-TextCNN model further improved accuracy to 0.869. A comparative analysis of the MuTCELM and ELM models, as shown in Table 4, reveals that while the ELM model performed satisfactorily with an accuracy of 0.919, the proposed MuTCELM outperformed all benchmark methods with an accuracy of 0.930. Compared to the best results of the baseline models, the accuracy of the ELM improved by 1.058%, while the proposed MuTCELM achieved a 7.01% increase. In terms of specific performance metrics, MuTCELM demonstrated superior classification results, achieving an accuracy of 0.930, which surpasses that of all other models, including the best baseline model (ELM) with 0.919. MuTCELM also achieved a precision of 0.925, a recall of 0.929, and an F1-macro score of 0.927, outperforming all baseline models. Overall, MuTCELM consistently outperformed baseline models and other models enhanced with Multi-TextCNN across accuracy, precision, recall, and F1-macro metrics, as illustrated in Fig. 3.

#### 4.1.2 Results on ALJ-news dataset

**Figure 4:**
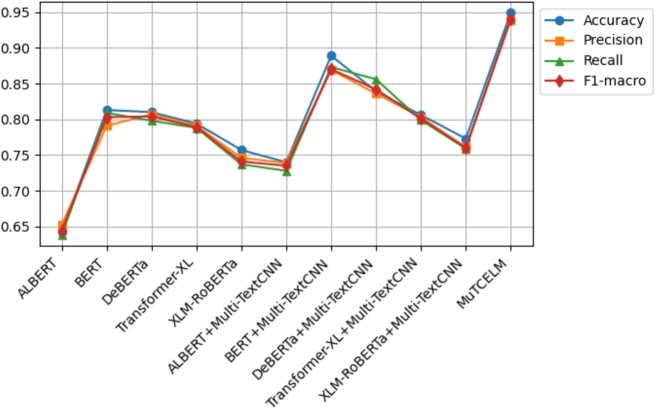
Summary of models performance on ALJ-News dataset.

Initially, a two-fold Grey Wolf Optimizer (GWO) within a wrapper feature selection method was applied to the ALJ-news text data for a downstream task, achieving an accuracy of 0.833 with the Naïve Bayes classifier [54]. As shown in Table 5, the proposed MuTCELM achieved the highest performance across all metrics, with an accuracy of 0.949. Compared to the ensemble without Multi-TextCNN, MuTCELM improved accuracy by 0.124 and by 0.209 when using the proposed Multi-TextCNN model. Specifically, MuTCELM outperformed baseline models in individual metrics: for accuracy, MuTCELM achieved 0.930, representing an increase of 0.251 over ALBERT, 0.158 over DeBERTa, and 0.167 over Transformer-XL. In terms of precision, MuTCELM improved by 0.251 over ALBERT, 0.111 over BERT, 0.175 over DeBERTa, and 0.140 over XLM-RoBERTa. For recall, MuTCELM demonstrated improvements of 0.268 over ALBERT, 0.175 over DeBERTa, 0.168 over Transformer-XL, and 0.140 over XLM-RoBERTa. Regarding F1-macro, MuTCELM achieved an increase of 0.260 over ALBERT, 0.107 over BERT, 0.166 over DeBERTa, and 0.134 over XLM-RoBERTa. These results clearly indicate that MuTCELM outperforms both baseline models and models enhanced with Multi-TextCNN across all performance metrics (accuracy, precision, recall, and F1-macro). The improvements are particularly significant in cases of initially lower performance, underscoring MuTCELM's effectiveness and robustness in enhancing model accuracy and reliability on the ALJ-news dataset. Overall, the performance of MuTCELM on this dataset, as depicted in Fig. 4, demonstrates its efficacy in Arabic text classification. Multi-TextCNN, in particular, excels in achieving a balanced performance across evaluation metrics.

**Table 5:** Results on ALJ-News dataset.

Type	Model	Accuracy	Precision	Recall	F1-macro	AVG
	ALBERT	0.643	0.653	0.639	0.645	0.645
	BERT	0.813	0.791	0.809	0.799	0.803
	DeBERTa	0.810	0.807	0.798	0.802	0.804
Baseline	Transformer-XL	0.794	0.785	0.791	0.788	0.789
	XLM-RoBERTa	0.757	0.746	0.737	0.741	0.745
	ELM	0.937	0.934	0.931	0.932	0.933

	ALBERT + Multi-TextCNN	0.740	0.739	0.728	0.733	0.735
	BERT + Multi-TextCNN	0.889	0.869	0.873	0.870	0.875
Effects of Multi-TextCNN	DeBERTa + Multi-TextCNN	0.839	0.831	0.829	0.830	0.832
	Transformer-XL + Multi-TextCNN	0.806	0.803	0.799	0.800	0.802
	XLM-RoBERTa + Multi-TextCNN	0.773	0.761	0.759	0.760	0.763
	**MuTCELM (Proposed)**	0.949	0.939	0.942	0.940	0.942

#### 4.1.3 Results on ArSarcasm-V2 dataset

**Figure 5:**
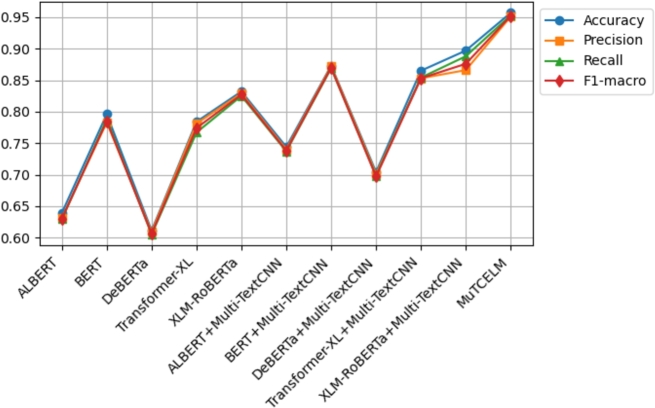
Summary of models performance on ArSarcasm-V2 dataset.

Mahdaouy et al. [58] achieved an accuracy score of 0.662 on the ArSarcasm-v2 dataset using a dual-task learning technique, while Touahri et al. (2021) [59] enhanced the modeling of sarcastic characteristics, resulting in an accuracy of 0.803. As shown in Table 6, we conducted several experiments with five different baseline models, including versions with and without the proposed Multi-TextCNN. The experimental results indicate that XLM-RoBERTa + Multi-TextCNN outperformed the original accuracy by 35.4%. In contrast, ELM achieved an accuracy of 0.948, while the proposed MuTCELM surpassed all benchmark models, achieving an accuracy of 0.957. These findings demonstrate that the Multi-TextCNN effectively captures semantic features from the ArSarcasm-V2 dataset compared to other baseline models. In terms of accuracy, ALBERT achieved a score of 0.639, BERT achieved 0.796, DeBERTa achieved 0.611, Transformer-XL achieved 0.784, and XLM-RoBERTa achieved 0.832. In contrast, MuTCELM attained an accuracy of 0.957, reflecting an improvement of 0.318, 0.346, 0.173, and 0.125 over ALBERT, DeBERTa, Transformer-XL, and XLM-RoBERTa, respectively. The influence of Multi-TextCNN is evident across all baseline models, as ALBERT + Multi-TextCNN reached 0.744, BERT + Multi-TextCNN reached 0.873, and Transformer-XL + Multi-TextCNN reached 0.865, marking a substantial enhancement compared to their original results. Regarding precision, ALBERT, BERT, DeBERTa, Transformer-XL, and XLM-RoBERTa achieved 0.631, 0.783, 0.609, 0.781, and 0.819, while MuTCELM achieved 0.951, indicating improvements of 0.320, 0.168, 0.342, 0.170, and 0.132, respectively. Although models incorporating Multi-TextCNN exhibited slight improvements, MuTCELM consistently demonstrated significant advancements. In terms of recall, MuTCELM improved results by increments of 0.324, 0.165, 0.348, 0.186, and 0.128, respectively. When compared to Multi-TextCNN-based models, MuTCELM showed improvements of 0.216, 0.084, 0.253, 0.099, and 0.077. For F1-macro, MuTCELM improved the results with increments of 0.322, 0.167, 0.346, 0.178, and 0.125, and, compared to Multi-TextCNN-based models, achieved improvements of 0.214, 0.082, 0.251, 0.099, and 0.076. These enhancements are particularly noteworthy when compared to baseline models, where the increases are more pronounced. This analysis demonstrates that the proposed Multi-TextCNN not only enhances standard transformer models but also significantly surpasses their individual performances. Overall, the performance of MuTCELM on the ArSarcasm-V2 dataset, as illustrated in Fig. 5, underscores its efficacy in text classification. In particular, Multi-TextCNN exhibits exceptional capability in achieving a balanced performance across the evaluation metrics.

**Table 6:** Results on ArSarcasm-V2 dataset.

Type	Model	Accuracy	Precision	Recall	F1-macro	AVG
	ALBERT	0.639	0.631	0.629	0.630	0.632
	BERT	0.796	0.783	0.788	0.785	0.788
	DeBERTa	0.611	0.609	0.605	0.606	0.607
Baseline	Transformer-XL	0.784	0.781	0.767	0.774	0.776
	XLM-RoBERTa	0.832	0.829	0.825	0.827	0.828
	ELM	0.948	0.944	0.937	0.940	0.942

	ALBERT + Multi-TextCNN	0.744	0.740	0.737	0.738	0.739
	BERT + Multi-TextCNN	0.873	0.872	0.869	0.870	0.871
Effects of Multi-TextCNN	DeBERTa + Multi-TextCNN	0.704	0.701	0.698	0.699	0.700
	Transformer-XL + Multi-TextCNN	0.865	0.853	0.854	0.853	0.856
	XLM-RoBERTa + Multi-TextCNN	0.897	0.866	0.888	0.876	0.881
	**MuTCELM (Proposed)**	0.957	0.951	0.953	0.952	0.953

#### 4.1.4 Results on Ewe dataset

**Figure 6:**
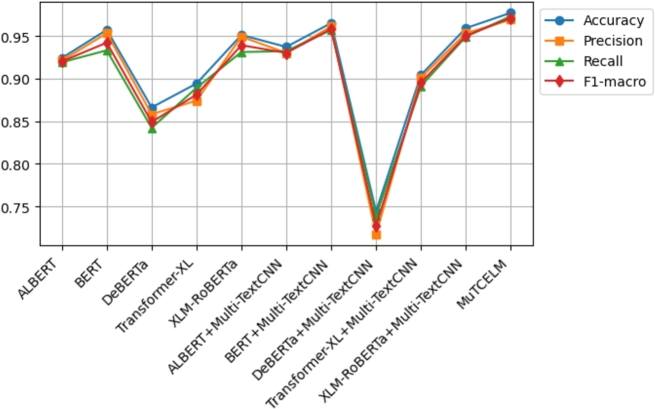
Summary of models performance on Ewe dataset.

According to Agbesi et al. [25], the initial experimental findings on the Ewe language indicated that the highest accuracy achieved was 0.862 using the fine-tuned EweBERT model. Subsequently, an adaptive convolutional-based technique led to an improved accuracy of 0.930 on the Ewe dataset [7]. In our experiments, we applied the Multi-TextCNN to the baseline model to evaluate its robustness compared to the proposed MuTCELM. As shown in Table 7, the ELM achieved an accuracy of 0.963, while the proposed MuTCELM attained an optimal accuracy of 0.977. This study examines MuTCELM's enhancements in specific performance metrics relative to both the baseline and Multi-TextCNN-enhanced models. In terms of accuracy, MuTCELM showed improvements of 0.053, 0.020, 0.111, 0.083, and 0.026 compared to the baseline models. Compared to the Multi-TextCNN-based models, MuTCELM achieved further gains of 0.040, 0.012, 0.233, 0.073, and 0.018, respectively. In precision, MuTCELM demonstrated increments of 0.048, 0.016, 0.111, 0.095, and 0.020 compared to the baseline and improvements of 0.039, 0.008, 0.238, 0.078, and 0.016 over the Multi-TextCNN-based models. For recall, MuTCELM achieved enhancements of 0.054, 0.040, 0.131, 0.104, and 0.032 compared to the baseline and outperformed the Multi-TextCNN-based models by 0.041, 0.016, 0.246, 0.078, and 0.024. In terms of F1-macro, MuTCELM showed improvements of 0.051, 0.039, 0.122, 0.090, and 0.032 over the baseline models and further recorded increases of 0.041, 0.013, 0.243, 0.076, and 0.021 compared to the Multi-TextCNN-based models. The results on the Ewe dataset clearly demonstrate that MuTCELM surpasses both the baseline and Multi-TextCNN-enhanced models across all performance metrics, including accuracy, precision, recall, and F1-macro. These improvements are particularly significant when compared to the baseline models, highlighting MuTCELM's effectiveness in enhancing model performance. Overall, the performance of MuTCELM on the Ewe dataset, as depicted in Fig. 6, demonstrates exceptional effectiveness in Ewe text classification, particularly in achieving a balanced performance across all evaluation metrics.

**Table 7:** Results on Ewe dataset.

Type	Model	Accuracy	Precision	Recall	F1-macro	AVG
	ALBERT	0.924	0.921	0.919	0.920	0.921
	BERT	0.957	0.953	0.933	0.942	0.946
	DeBERTa	0.866	0.858	0.842	0.849	0.853
Baseline	Transformer-XL	0.894	0.874	0.889	0.881	0.884
	XLM-RoBERTa	0.951	0.949	0.931	0.939	0.942
	ELM	0.963	0.958	0.950	0.953	0.956

	ALBERT + Multi-TextCNN	0.937	0.930	0.932	0.930	0.932
	BERT + Multi-TextCNN	0.965	0.961	0.957	0.958	0.960
Effects of Multi-TextCNN	DeBERTa + Multi-TextCNN	0.744	0.717	0.739	0.727	0.731
	Transformer-XL + Multi-TextCNN	0.904	0.901	0.891	0.895	0.897
	XLM-RoBERTa + Multi-TextCNN	0.959	0.953	0.949	0.950	0.952
	**MuTCELM (Proposed)**	0.977	0.969	0.973	0.971	0.973

#### 4.1.5 Results on Urdu corpus

**Figure 7:**
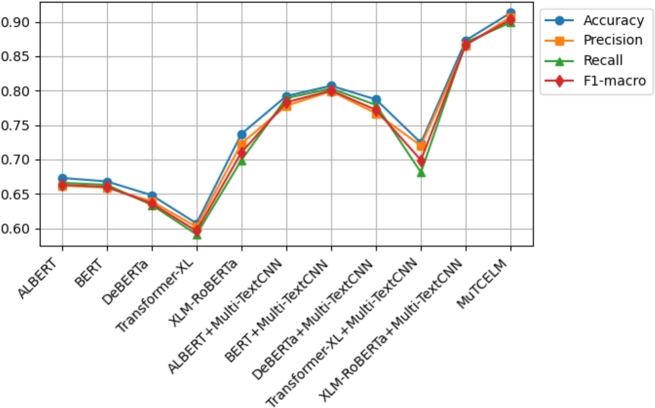
Summary of models performance on Urdu corpus.

The initial result obtained on the Urdu corpus was 0.760 [56]. However, in our study, we examined the efficiency of the proposed Multi-TextCNN on each model and compared their performance to MuTCELM. As presented in Table 8, the findings indicate that the proposed MuTCELM outperformed all compared models, achieving an accuracy of 0.913. This study systematically compares MuTCELM's improvements in specific performance metrics to those of both baseline and Multi-TextCNN-based models. In terms of accuracy, MuTCELM demonstrated improvements of 0.240, 0.245, 0.265, 0.306, and 0.176 over the baseline models. Compared to Multi-TextCNN-based models, MuTCELM recorded increases of 0.121, 0.106, 0.126, 0.189, and 0.040, respectively. Regarding precision, MuTCELM improved results by 0.245, 0.248, 0.268, 0.305, and 0.184 on the baseline models and showed gains of 0.129, 0.108, 0.140, 0.206, and 0.041 compared to Multi-TextCNN-based models. For the recall metric, MuTCELM exhibited improvements of 0.233, 0.236, 0.265, 0.308, and 0.200 over the baseline and 0.121, 0.096, 0.120, 0.208, and 0.029 compared to Multi-TextCNN-based models. In terms of F1-macro, MuTCELM recorded enhancements of 0.240, 0.243, 0.267, 0.307, and 0.193, respectively, and an average improvement of 0.116 when compared to the Multi-TextCNN-based models. These results underscore MuTCELM's effectiveness in enhancing model performance, demonstrating significant improvements, and highlighting its superior performance. Compared to the baseline models, MuTCELM's performance on the Urdu corpus, as depicted in Fig. 7, illustrates its exceptional effectiveness.

**Table 8:** Results on Urdu corpus.

Type	Model	Accuracy	Precision	Recall	F1-macro	AVG
	ALBERT	0.673	0.662	0.666	0.663	0.666
	BERT	0.668	0.659	0.663	0.660	0.662
	DeBERTa	0.648	0.639	0.634	0.636	0.639
Baseline	Transformer-XL	0.607	0.602	0.591	0.596	0.599
	XLM-RoBERTa	0.737	0.723	0.699	0.710	0.717
	ELM	0.891	0.883	0.879	0.880	0.883

	ALBERT + Multi-TextCNN	0.792	0.778	0.789	0.783	0.785
	BERT + Multi-TextCNN	0.807	0.799	0.803	0.800	0.802
Effects of Multi-TextCNN	DeBERTa + Multi-TextCNN	0.787	0.767	0.779	0.772	0.776
	Transformer-XL + Multi-TextCNN	0.724	0.720	0.681	0.699	0.706
	XLM-RoBERTa + Multi-TextCNN	0.873	0.866	0.870	0.867	0.869
	**MuTCELM (Proposed)**	0.913	0.907	0.899	0.903	0.906

### 4.2 Statistical analysis

A statistical analysis was conducted based on the performance of each model across all datasets. To achieve this, the study computed the confidence intervals and conducted statistical significance tests using the scores obtained in the evaluation metrics. Confidence intervals provide a range within which the true accuracy is expected to lie with a 95% confidence level. Table 9 presents the confidence intervals for each model. According to the table, MuTCELM consistently outperforms all baseline models across all datasets, as indicated by the higher confidence interval ranges, which reflect superior performance and stability. Compared to the baseline models, MuTCELM significantly improves performance with a narrower interval, suggesting more robust stability and reliability. The effectiveness of Multi-TextCNN in enhancing MuTCELM's performance is particularly evident when compared to the respective baseline models, underscoring its robustness across different datasets. Furthermore, the non-overlapping confidence intervals between MuTCELM and the other baseline models indicate statistically significant differences, demonstrating that MuTCELM's performance improvements are not due to random chance but are statistically meaningful. This evidence underscores the robustness and superiority of MuTCELM in various classification tasks. Furthermore, we conduct a statistical significance test to compare three sets of models: Baseline models versus Multi-TextCNN models, Multi-TextCNN models versus MuTCELM, and MuTCELM versus Baseline models across various datasets. The results are presented in Table 10. For the comparison between Baseline and Multi-TextCNN models, the negative t-statistic on the AJGT and ArSarcasm-V2 datasets indicates that the Multi-TextCNN models perform significantly better than the baseline models, with a p-value less than 0.05, signifying statistical significance. However, the p-value for ALJ-news is slightly above 0.05, indicating that the result is not statistically significant at the 5% level. For the Ewe dataset, the positive t-statistic suggests minimal improvement in Multi-TextCNN models over baseline models, with a p-value of 0.5631. Similarly, when comparing Multi-TextCNN models with MuTCELM, MuTCELM significantly outperformed the Multi-TextCNN models, as indicated by very low p-values on the ALJ-news, AJGT, ArSarcasm-V2, and Urdu datasets, reflecting high statistical significance. However, the p-value for the Ewe text indicates no statistically significant difference between MuTCELM and Multi-TextCNN models. Lastly, the consistently low p-values across each dataset demonstrate that MuTCELM consistently outperforms the baseline models, indicating high statistical significance. In summary, MuTCELM shows statistically significant improvements over both baseline and Multi-TextCNN models across these datasets. However, in the Ewe dataset, the improvements were not statistically significant, suggesting potential variability in the text. Overall, MuTCELM's consistent outperformance across these datasets underscores its robustness and effectiveness in enhancing text classification tasks.

**Table 9:** Confidence Interval for MuTCELM and baseline models.

Model	AJGT	ALJ-News	ArSarcasm-V2	Ewe	Urdu Corpus
ALBERT	0.668, 0.681	0.639, 0.650	0.627, 0.641	0.918, 0.923	0.660, 0.686
BERT	0.816, 0.828	0.794, 0.812	0.779, 0.791	0.935, 0.956	0.655, 0.673
DeBERTa	0.761, 0.775	0.799, 0.809	0.607, 0.615	0.843, 0.864	0.628, 0.654
Transformer-XL	0.758, 0.765	0.786, 0.791	0.772, 0.782	0.875, 0.893	0.588, 0.610
XLM-RoBERTa	0.790, 0.804	0.732, 0.749	0.825, 0.835	0.939, 0.950	0.708, 0.738
ELM	0.915, 0.923	0.932, 0.941	0.939, 0.949	0.950, 0.961	0.875, 0.897
ALBERT+Multi-TextCNN	0.787, 0.801	0.729, 0.740	0.737, 0.749	0.929, 0.935	0.776, 0.798
BERT+Multi-TextCNN	0.838, 0.848	0.861, 0.878	0.868, 0.878	0.956, 0.963	0.794, 0.814
DeBERTa+Multi-TextCNN	0.856, 0.872	0.832, 0.851	0.696, 0.708	0.719, 0.742	0.758, 0.793
Transformer-XL+Multi-TextCNN	0.810, 0.824	0.797, 0.804	0.856, 0.864	0.892, 0.903	0.704, 0.728
XLM-RoBERTa+Multi-TextCNN	0.833, 0.847	0.752, 0.767	0.860, 0.880	0.948, 0.957	0.861, 0.879
**MuTCELM(Proposed)**	0.922, 0.936	0.935, 0.944	0.950, 0.964	0.969, 0.975	0.899, 0.917

**Table 10:** Statistical significance test for MuTCELM and baseline models.

Comparison	Test	AJGT	ALJ-News	ArSarcasm-V2	Ewe	Urdu Corpus
Baseline vs Multi-TextCNN models	t-statistic	-3.6884	-2.6905	-3.6289	0.6296	-26.3177
	p-value	0.0210	0.0546	0.0221	0.5631	1.2387

Multi-TextCNN models vs MuTCELM	t-statistic	-7.5642	-5.4002	5.0166	-1.8398	-4.8906
	p-value	0.0016	0.0056	0.0074	0.1396	0.0080

MuTCELM vs Baseline	t-statistic	6.1849	5.8538	-6.5387	3.4045	11.6823
	p-value	0.0034	0.0042	0.0028	0.0271	0.0003

### 4.3 Ablation study

**Figure 8:**
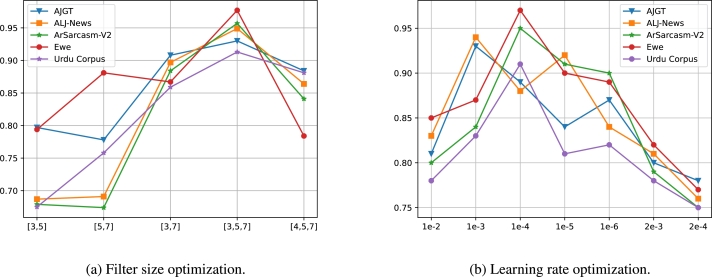
Impact of hyperparameter variation on classification accuracy across datasets.

The impact of hyperparameter variation on classification accuracy, including dynamic filter sizes ([3,5], [5,7], [3,7], [3,5,7], and [4,5,7]) and diverse learning rates (1e-2, 1e-3, 1e-4, 1e-5, 1e-6, 2e-3, and 2e-4), were examined with the proposed MuTCELM as shown in Fig. 8. The filter size in this study influences the local features and textual patterns captured by the CNN, while the learning rate affects the overall performance of the classification model. A 5-fold cross-validation strategy was employed to evaluate the performance of each parameter. As illustrated in Fig. 8, the impact of different filter sizes on the accuracy of MuTCELM was assessed for each dataset, while various learning rates were tested to determine the optimal rate for MuTCELM's efficiency and robustness.

The results indicate that the choice of filter size significantly influences the diversity and generalization of the proposed MuTCELM. It was observed that as the number of feature maps decreases, classification accuracy drops, but it improves with an increase in feature maps. As shown in Fig. 8(a), different filter sizes impact the accuracy of each dataset when using a CNN model. For instance, with a filter size of [3,5], the AJGT dataset achieved a moderate accuracy of 0.797, suggesting that while this filter size captures relevant features, it is not optimal. The ALJ-news dataset recorded a lower accuracy of 0.687, indicating that this filter size is less effective for this dataset, while the ArSarcasm-V2 dataset also achieved a lower accuracy of 0.679, showing limited effectiveness. The Ewe dataset achieved an accuracy of 0.794, comparable to the AJGT dataset. However, the Urdu corpus recorded the lowest accuracy among the datasets, indicating relatively weak performance with this filter size. For the filter size of [5,7], the AJGT dataset achieved a slightly lower accuracy of 0.778 compared to [3,5], suggesting this filter size is less suitable. The ALJ-news dataset saw a marginal improvement to 0.691, but this is still low. Conversely, the Ewe dataset showed significant improvement with an accuracy of 0.881, indicating that this filter size is adequate for this dataset. The Urdu corpus also improved to 0.758, while the ArSarcasm-V2 dataset recorded a slight decrease to 0.674, indicating reduced effectiveness. With a filter size of [3,7], the AJGT dataset achieved a substantial improvement, reaching an accuracy of 0.908, indicating that this filter size captures more relevant features. The ALJ-news dataset also achieved high accuracy at 0.897, suggesting strong effectiveness, while the ArSarcasm-V2 and Urdu datasets showed significant improvements with accuracies of 0.884 and 0.859, respectively. However, the Ewe dataset saw a slight decrease from the previous filter size. The proposed filter size of [3,5,7] yielded the highest accuracies across multiple datasets, with the AJGT dataset achieving 0.930, indicating that this combination captures the most relevant features. Similarly, the ALJ-news and ArSarcasm-V2 datasets recorded high accuracies of 0.864 and 0.957, respectively, making this filter size optimal for Arabic text. The Ewe dataset achieved the highest accuracy of 0.977 among all datasets and filter sizes, demonstrating the effectiveness of this combination. However, with the filter size of [4,5,7], both the AJGT and ALJ-news datasets saw a decrease in accuracy, indicating reduced effectiveness. The ArSarcasm-V2 dataset also experienced a significant decrease, while the Ewe dataset showed a large drop in accuracy to 0.784, indicating poor performance. The Urdu corpus achieved an accuracy of 0.881; though slightly reduced from its peak, it was still relatively high. Overall, the filter size [3,5] exhibited moderate effectiveness but was generally less optimal compared to larger combinations. The filter size [3,7] performed well, significantly improving accuracy over smaller combinations but being slightly less effective than [3,5,7]. Filter sizes [5,7] and [4,5,7] produced mixed results; for instance, the Ewe dataset benefited from [5,7], but these filter sizes were generally less effective than [3,5,7] and [3,7]. Ultimately, the results demonstrate that the filter size [3,5,7] is the most effective combination for the AJGT, ALJ-news, ArSarcasm-V2, Ewe, and Urdu datasets, providing the highest accuracy. Additionally, the choice of filter size significantly impacts the performance of CNN models, as observed. Moreover, increasing the filter size enhances model complexity and extends training time. Data-specific tuning remains crucial, as other datasets might respond differently to various filter sizes, but [3,5,7] serves as a robust starting point for achieving high accuracy in this study.

The proposed filter size was further evaluated with various learning rates to determine the most effective rate, as depicted in Fig. 8(b). According to the figure, a learning rate of 1e-2 resulted in the AJGT and ArSarcasm-V2 datasets achieving accuracies of 0.81 and 0.80, respectively, indicating relatively strong performance. The ALJ-news dataset recorded a moderate accuracy of 0.83, suggesting that while the model is learning effectively, it is not doing so optimally. The Ewe dataset demonstrated a higher accuracy at this rate, indicating good learning capability, whereas the Urdu corpus recorded the lowest accuracy, suggesting less effective learning. With a learning rate of 1e-3, significant improvements were observed in the AJGT, Ewe, and Urdu datasets, indicating that this rate is optimal and effective for these datasets. The ArSarcasm-V2 dataset also showed a slight improvement compared to 1e-2. At 1e-4, the AJGT and ALJ-news datasets experienced a slight decrease in accuracy, while the ArSarcasm-V2 dataset achieved a significant improvement, marking the highest accuracy among all rates for this dataset. This suggests that 1e-4 is particularly effective for the ArSarcasm-V2 dataset, resulting in a high accuracy of 0.95. Similarly, the Ewe and Urdu datasets recorded high accuracies of 0.97 and 0.91, respectively, indicating that this rate is also optimal for these texts. At a learning rate of 1e-5, the AJGT, ArSarcasm-V2, Ewe, and Urdu datasets exhibited a decline in accuracy compared to previous rates, indicating slower and less optimal learning. However, the ALJ-news dataset showed a slight improvement, though it still did not have an optimal learning rate. The 1e-6 rate led to moderate accuracy for the AJGT dataset, reflecting slow learning. The decrease in the ALJ-news dataset at this rate indicates less effective learning. For the 2e-3 rate, the AJGT dataset achieved an accuracy of 0.80, reflecting a decline and suggesting that this rate is suboptimal. The ALJ-news, Urdu, and Ewe datasets also showed suboptimal learning, while the ArSarcasm-V2 dataset achieved an accuracy of 0.79, indicating less effective learning. Finally, with the 2e-4 rate, all datasets recorded their lowest accuracy, indicating that this rate is ineffective for learning across these datasets. In this experiment, the 2e-3 and 2e-4 rates consistently yielded lower accuracies, marking them as suboptimal learning rates. The rates of 1e-2, 1e-5, and 1e-6 exhibited moderate to high accuracy but were not as effective as the optimal learning rates. The 1e-4 rate demonstrated high effectiveness, particularly for the ArSarcasm-V2 and Ewe datasets. However, the 1e-3 learning rate proved to be the most effective, achieving the highest accuracy across the AJGT, ALJ-news, ArSarcasm-V2, and Ewe datasets, making it generally the most effective learning rate, especially with a dropout rate of 0.5. This experiment highlights the significant impact of learning rate selection on the training of text classification models. It was observed that excessively high learning rates can lead to model instability and divergence, while excessively low rates result in slow convergence.

Table 11 provides a comprehensive summary of the accuracy results across various benchmark datasets, including the performance of several model combinations. The table also highlights the enhanced accuracy of specific baseline models through underlined scores and presents each model's ranking alongside the highest accuracy score, shown in round brackets. The findings indicate that MuTCELM effectively integrates the strengths of Multi-TextCNN, offering a robust and reliable solution for text classification tasks across different languages. This results in improved accuracy and reliability. Table 12 further details the evaluation results of the proposed MuTCELM, including the standard deviation and mean value for each of the five datasets. For the AJGT dataset, a standard deviation of 0.0005 is reported, indicating minimal variability across multiple evaluations. This suggests that MuTCELM consistently performs well with this dataset, exhibiting slight variation in outcomes. The standard deviation of 0.0016 achieved on the ALJ-news dataset reflects slightly higher variability compared to the AJGT dataset, yet it still demonstrates relatively stable performance. Similarly, the ArSarcasm-V2 dataset's standard deviation of 0.0005 and the Ewe and Urdu datasets' standard deviation of 0.0003 indicate minimal variability in performance. This demonstrates that MuTCELM consistently performs well across multiple evaluations on these datasets. Overall, these standard deviation results highlight MuTCELM's remarkable consistency and stability in performance across all the datasets examined. Such low variability suggests that MuTCELM is a robust and reliable model for text classification tasks across diverse datasets, including Ewe, Arabic, and Urdu corpora. These results underscore MuTCELM's ability to generalize effectively and maintain consistent performance across various textual datasets. In addition to these comparisons, MuTCELM's performance was evaluated against well-known ensemble techniques using the ArSarcasm-V2 and AJGT datasets. As shown in Table 13, MuTCELM demonstrated superior robustness on the ArSarcasm-V2 dataset compared to models proposed by Mahdaouy et al. [58], Song et al. [49], and Mohamed et al. [51]. Specifically, MuTCELM achieved an accuracy of 0.957 and an F1-macro score of 0.952, whereas the other models reported lower F1-macro scores of 0.662, 0.657, and 0.672, respectively. Similarly, on the AJGT dataset, MuTCELM outperformed models by Saleh et al. [60] and Mohammed and Kora [3], achieving an accuracy of 0.930, further indicating its robustness and effectiveness in text classification. Moreover, when compared with the bagging ensemble strategy, MuTCELM proved to be more effective in classifying the Arabic, Ewe, and Urdu datasets. Although the stacking ensemble strategy performed relatively well, it was not as optimal as MuTCELM in terms of classification accuracy. Therefore, based on the numerical results obtained, this study confidently identifies MuTCELM as an optimal model for classifying the AJGT, ALJ-news, ArSarcasm-V2, Ewe, and Urdu datasets, with accuracies of 0.930, 0.949, 0.957, 0.977, and 0.913, respectively. Overall, MuTCELM ranks first on the Ewe dataset with an accuracy of 0.977, followed by the ArSarcasm-V2, ALJ-news, AJGT, and Urdu datasets. The performance of MuTCELM can be attributed to the complementary strengths of the five sub-classifiers. They contributed significantly to capturing syntactic nuances and were crucial for understanding semantic relationships. This diversity in feature extraction allowed MuTCELM to achieve superior classification results across different datasets.

**Table 11:** Summary of the accuracy of all models.

Model	AJGT	ALJ-News	ArSarcasm-V2	Ewe	Urdu Corpus
ALBERT	0.679	0.643	0.639	0.924	0.673
BERT	0.831(5)	0.813(5)	0.796	0.957(5)	0.668
DeBERTa	0.772	0.810	0.611	0.866	0.648
Transformer-XL	0.763	0.794	0.784	0.894	0.607
XLM-RoBERTa	0.806	0.757	0.832(5)	0.951	0.737(5)

ALBERT + BERT	0.797	0.648	0.695	0.798	0.712
ALBERT + DeBERTa	0.778	0.691	0.648	0.895(4)	0.687
ALBERT + Transformer-XL	0.884(4)	0.797	0.757	0.874	0.617
ALBERT + XLM-RoBERTa	0.730	0.655	0.574	0.913	0.647
BERT + DeBERTa	0.808	0.824(4)	0.674	0.875	0.687
BERT + Transformer-XL	0.802	0.771	0.614	0.825	0.815
BERT + XLM-RoBERTa	0.817	0.818	0.789	0.895	0.748
DeBERTa + Transformer-XL	0.648	0.731	0.728	0.860	0.795
DeBERTa + XLM-RoBERTa	0.874	0.799	0.814	0.853	0.825(4)
Transformer-XL + XLM-RoBERTa	0.775	0.769	0.831(4)	0.884	0.818

ALBERT+BERT+DeBERTa	0.851	0.794	0.837	0.908	0.712
BERT+Transformer-XL+XLM-RoBERTa	0.866	0.922(3)	0.799	0.923	0.815
DeBERTa+XLM-RoBERTa+ALBERT	0.814	0.849	0.899(3)	0.913	0.825(3)
Transformer-XL+XLM-RoBERTa+DeBERTa	0.905(3)	0.884	0.879	0.891	0.818
Transformer-XL+BERT+DeBERTa	0.855	0.799	0.887	0.932(3)	0.818

ALBERT+BERT+DeBERTa+ Transformer-XL	0.847	0.804	0.835	0.945	0.842
BERT+Transformer-XL+XLM-RoBERTa+ALBERT	0.919(2)	0.937(2)	0.788	0.939	0.899(2)
DeBERTa+XLM-RoBERTa+BERT+ALBERT	0.884	0.836	0.891	0.933	0.857
Transformer-XL+BERT+XLM-RoBERTa+DeBERTa	0.911	0.897	0.901(2)	0.955(2)	0.888
**MuTCELM (Proposed)**	0.930(1)	0.949(1)	0.957(1)	0.977(1)	0.913(1)

**Table 12:** Summary of the accuracy of all models.

Fold	AJGT	ALJ-News	ArSarcasm-V2	Ewe	Urdu Corpus
Fold 1	0.9300	0.9477	0.9559	0.9769	0.9129
Fold 2	0.9308	0.9492	0.9569	0.9767	0.9133
Fold 3	0.9305	0.9481	0.9570	0.9771	0.9127
Fold 4	0.9296	0.9494	0.9563	0.9766	0.9134
Fold 5	0.9303	0.9455	0.9571	0.9773	0.9132

Mean (*μ*)	0.9302	0.9480	0.9566	0.9769	0.9131
Standard deviation (*σ*)	0.0005	0.0016	0.0005	0.0003	0.0003

**Table 13:** Result comparison of MuTCELM, benchmark methods, and ensemble techniques.

Dataset	Model	F1-macro	Accuracy
	El Mahdaouy et al., (2021) [58] (MTL-approach)	0.662	−
ArSarcasm-V2	Song et al., (2021) [49] (Deep ensemble approach)	0.657	−
	Mohamed et al., (2023) [51] (XLM-T+MARBERT)	0.672	−
	Bagging	0.939	0.943
	Stacking	0.946	0.949
	**MuTCELM (Proposed)**	0.952	0.957

	Mohammed et al., (2022) [3]	−	0.88
AJGT	Saleh et al., (2022) [60] (Stacking LR)	0.86	0.86
	Bagging	0.897	0.900
	Stacking	0.925	0.927
	**MuTCELM (Proposed)**	0.927	0.930

	Bagging	0.929	0.935
ALJ-News	Stacking	0.933	0.938
	**MuTCELM (Proposed)**	0.940	0.949

	Bagging	0.949	0.954
Ewe	Stacking	0.970	0.974
	**MuTCELM (Proposed)**	0.971	0.977

	Bagging	0.863	0.899
Urdu Corpus	Stacking	0.905	0.910
	**MuTCELM (Proposed)**	0.903	0.913

### 4.4 Discussion

**Figure 9:**
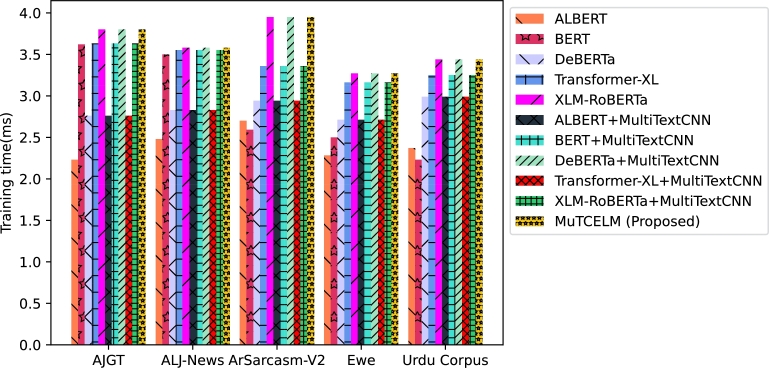
Training time.

**Figure 10:**
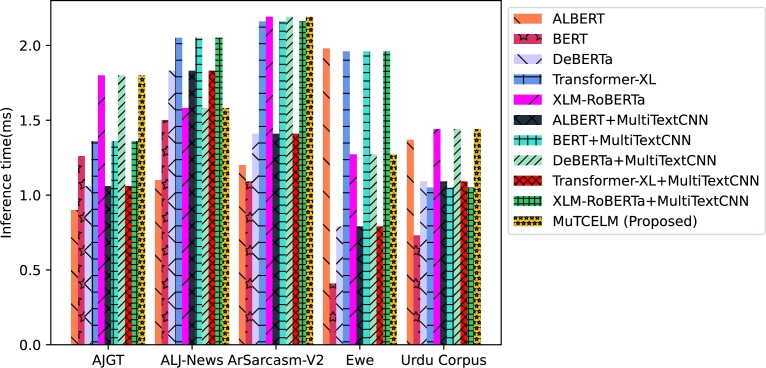
Inference time.

The results across all benchmark datasets demonstrate that the proposed MuTCELM achieves optimal classification performance without requiring additional hyperparameter adjustments. This study indicates that integrating the proposed Multi-TextCNN with probability distributions of class predictions from baseline models enhances ensemble performance compared to using standalone models and class label predictions. Overall, MuTCELM is a practical approach for improving classification accuracy and robustness in text classification tasks. It shows particular strength in sentiment analysis, news classification, and fake news detection. Models such as ALBERT, DEBERTa, Transformer-XL, and XLM-RoBERTa contribute distinct advantages to this ensemble. ALBERT emphasizes parameter efficiency and has a smaller memory footprint compared to BERT. DEBERTa captures long-range dependencies efficiently, while Transformer-XL manages longer texts. XLM-RoBERTa, trained on a multilingual corpus, is well-suited for multilingual text classification tasks. Combining these models allows the ensemble to capture various linguistic features and patterns, enhancing robustness across different domains (e.g., sentiment analysis, fake news detection, and news classification) and languages, including Arabic, Ewe, and Urdu. The deployment of a weighted voting ensemble technique ensures that the final prediction is not overly reliant on any single sub-classifier but rather integrates the strengths of multiple sub-classifiers, leading to more robust and accurate predictions. The inclusion of diverse sub-classifiers enhances overall performance while mitigating the risk of overfitting. Fig. 9 and Fig. 10 illustrate the training and inference times for MuTCELM and baseline models. The results reveal that computational efficiency and model performance are not mutually exclusive. While ALBERT and other baseline models offer moderate training and inference times on each dataset, they do not match MuTCELM's effectiveness. The incorporation of Multi-TextCNN layers increases both training and inference times due to additional computational overhead; however, this complexity is justified by the enhanced performance. MuTCELM shows the highest training times across each dataset, underscoring the computational cost associated with achieving state-of-the-art performance. Despite this, the inference time difference among models is less pronounced than the training time difference, suggesting that MuTCELM is suitable for real-time applications, provided that training time is not a constraint.

**Figure 11:**
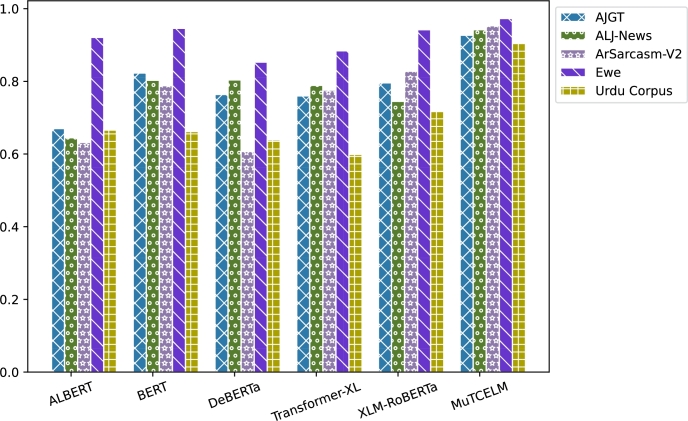
Average performance of MuTCELM and baseline models on each dataset.

In summary, the combination of kernel sizes [3, 5, 7] in MuTCELM facilitates multi-scale feature extraction, enabling the model to capture features at varying levels of granularity simultaneously. This leads to a richer and more comprehensive feature set for classification, enhancing the model's robustness and generalization capabilities. The experimental results confirm that models employing multiple kernel sizes outperform those using a single kernel size due to their improved ability to capture a wide range of patterns and dependencies, resulting in more accurate and reliable classification outcomes, as depicted in Fig. 11. The performance variations observed between the Ewe, Arabic, and Urdu datasets can be attributed to data-specific characteristics, which highlights the importance of considering linguistic diversity in model training. However, MuTCELM's dependency on data remains a notable challenge. Addressing this limitation through techniques such as efficient fine-tuning, transfer learning, and robust data augmentation can further enhance the model's applicability and performance. Additionally, leveraging sufficient GPUs and specialized hardware (e.g., TPUs) can help mitigate the computational costs involved.

## 5 Conclusion

Previous approaches utilizing machine learning and deep learning for text classification tasks focused on extracting textual features; however, these methods often resulted in sub-optimal performance due to their limited ability to effectively capture necessary features. In this study, we proposed MuTCELM, a novel Multi-TextCNN-based Ensemble Learning Model optimized for text classification tasks across multiple languages. Our approach integrates the strengths of five sub-classifiers, each specializing in distinct linguistic features, to enhance the overall performance of the ensemble model. Through comprehensive experiments on datasets in languages such as Ewe, Arabic, and Urdu, MuTCELM demonstrated superior classification accuracy, precision, recall, and F1-macro scores compared to existing methods. The key contributions of this work include the successful implementation of an ensemble learning framework that leverages deep learning models for multi-lingual text classification and the optimization techniques applied to enhance model performance. Additionally, the results underline the importance of model diversity within the ensemble, which contributes to a more robust and accurate classification system. Future work will focus on expanding the application of MuTCELM to a broader range of languages and domains, as well as exploring the integration of additional features such as contextual embeddings and transfer learning to further improve classification accuracy. The promising results obtained in this study suggest that MuTCELM has significant potential for real-world applications, particularly in areas requiring high accuracy and efficiency in processing and analyzing multilingual text data.

## CRediT authorship contribution statement

**Victor Kwaku Agbesi:** Writing – review & editing, Writing – original draft, Methodology, Formal analysis, Data curation, Conceptualization. **Wenyu Chen:** Supervision, Resources, Project administration, Methodology, Investigation. **Sophyani Banaamwini Yussif:** Visualization, Validation, Software, Conceptualization. **Chiagoziem C. Ukwuoma:** Writing – review & editing, Validation, Investigation, Formal analysis. **Yeong Hyeon Gu:** Visualization, Software, Project administration, Investigation, Funding acquisition. **Mugahed A. Al-antari:** Writing – review & editing, Project administration, Methodology, Investigation, Funding acquisition.

## Declaration of Competing Interest

The authors declare that they have no known competing financial interests or personal relationships that could have appeared to influence the work reported in this paper.

## Data Availability

Has data associated with your study been deposited into a publicly available repository? No, the datasets used are included in the article/supp. material/referenced in the manuscript.

## Appendix A Supplementary data

Supplementary data to this article are publicly available and freely accessible. They can be downloaded from the following database: https://web.eecs.umich.edu/~mihalcea/downloads.html##FakeNews (Urdu corpus), https://github.com/iabufarha/ArSarcasm-v2 (ArSarcasm-V2 dataset), AJGT dataset can be found https://github.com/komari6/Arabic-twitter-corpus-AJGT, the Ewe dataset can be downloaded from https://github.com/VictorAgbesi/Ewe-News-Dataset, and the Alj-News dataset can be downloaded via https://github.com/yalhag1/Alj-News-Arabic-text-classification-dataset. This study is implemented using the Python programming language in the PyTorch programming environment. However, the codes associated with this study will be made available after publication.
